# Embedding risk monitoring in infectious disease surveillance for timely and effective outbreak prevention and control

**DOI:** 10.1136/bmjgh-2024-016870

**Published:** 2025-02-17

**Authors:** Brecht Ingelbeen, Esther van Kleef, Placide Mbala, Kostas Danis, Ivalda Macicame, Niel Hens, Eveline Cleynen, Marianne A B van der Sande

**Affiliations:** 1 Julius Center for Health Sciences and Primary Care, University Medical Center Utrecht, Utrecht, Netherlands; 2 Institute of Tropical Medicine, Antwerpen, Belgium; 3 Nuffield Department of Medicine, University of Oxford, Oxford, UK; 4 Institut National de Recherche Biomédicale, Kinshasa, Democratic Republic of the Congo; 5 Independent scholar, Stockholm, Sweden; 6 Instituto Nacional de Saúde, Maputo, Mozambique; 7 Data Science Institute, Hasselt University, Hasselt, Belgium; 8 Sciensano, Brussel, Belgium

**Keywords:** Infections, diseases, disorders, injuries, Control strategies, Epidemiology, Decision Making

## Abstract

Epidemic intelligence efforts aim to predict, timely detect and assess (re-)emerging pathogens, guide and evaluate infectious disease prevention or control. We emphasise the underused potential of integrating the monitoring of risks related to exposure, disease or death, particularly in settings where limited diagnostic capacity and access to healthcare hamper timely prevention/control measures. Monitoring One Health exposures, human behaviour, immunity, comorbidities, uptake of control measures or pathogen characteristics can complement facility-based surveillance in generating signals of imminent or ongoing outbreaks, and in targeting preventive/control interventions or epidemic preparedness to high-risk areas or subpopulations. Low-cost risk data sources include electronic medical records, existing household/patient/environmental surveys, Health and Demographic Surveillance Systems, medicine distribution and programmatic data. Public health authorities need to identify and prioritise risk data that effectively fill gaps in intelligence that facility-based surveillance can not timely or accurately answer, determine indicators to generate from the data, ensure data availability, regular analysis and dissemination.

**SUMMARY BOX**

- Traditional infectious disease surveillance consisting of facility-based reporting of cases, often delays outbreak response due to disease progression, healthcare seeking, diagnostic and reporting lags.
- Monitoring of different risks—such as exposure proxies (e.g., repeated environmental sampling), pathogen characteristics (e.g., resistance genes), and human behavior (e.g., mobility data)—can provide real-time insights into outbreak potential, supplementing case-based surveillance. WHO advocated for the integration of threat, hazard and vulnerability data as part of surveillance.
- Health and demographic surveillance systems, repeated population (sero-)surveys, mobility, medical records, medicine use and health program data offer cost-effective alternatives to assess susceptibility, transmission patterns, and intervention effectiveness beyond facility-based case reporting.
- Advances in diagnostics, digital tools, and data interoperability and linkage can speed up outbreak detection, allow identifying at risk populations, and facilitate cross-sectoral collaboration for effective surveillance and timely public health responses.

## Introduction

**Figure 1:**
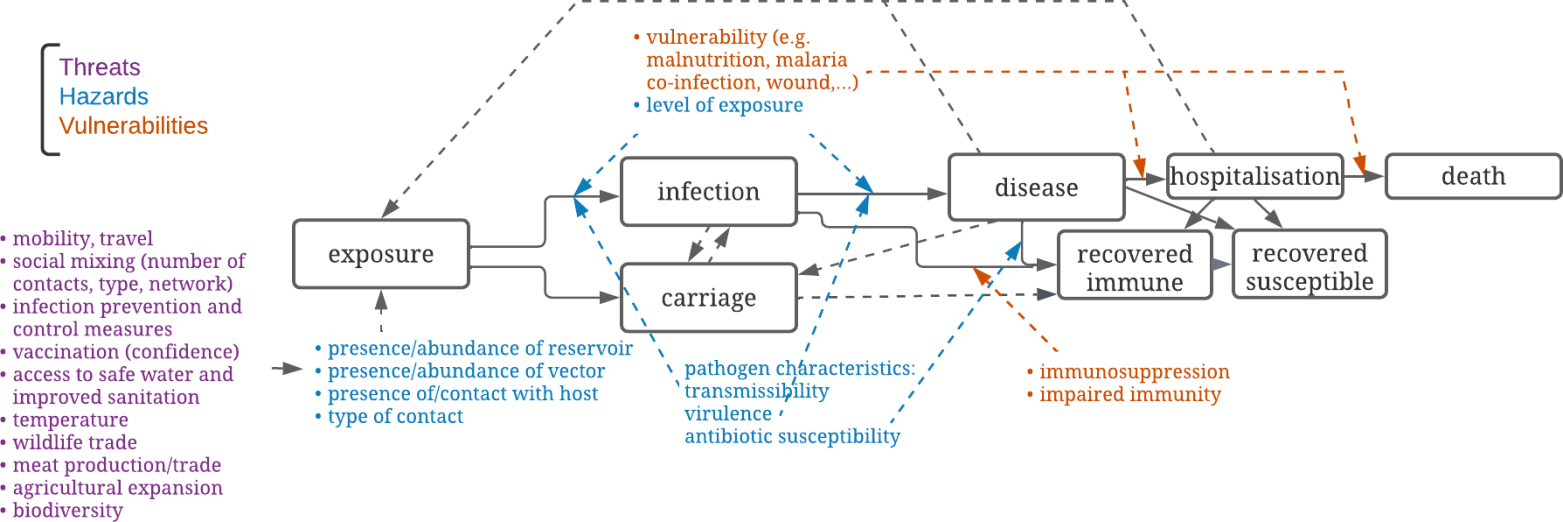
Stages of disease progression (black) with risk of exposure, infection, disease and disease progression: threats (purple), hazards (blue) and vulnerabilities (orange). Carriage is the ongoing colonisation of skin, genital mucosa, respiratory or gastrointestinal tract. Distinguishing the various stages reflects the main structure of compartmental mathematical models of many infectious diseases, illustrating the complexity of risk surveillance and understanding transmission during an outbreak.

 The frequency of emerging infectious disease outbreaks, the size of outbreaks and the proportion of outbreaks with antimicrobial-resistant (AMR) pathogens have been increasing for decades as a result of increased mobility, population density, agricultural expansion, meat production, wildlife trade and climate change.^1^,^8^ Effective infectious disease surveillance is needed to enable early warning and timely prevention and control interventions. Historically surveillance relied largely on healthcare facility-based reporting of laboratory-confirmed infections, clinical disease, hospital admissions or deaths—reflecting different stages of disease progression (figure 1). Facility-based case reports are, however, limited in guiding infections: delayed by the time lag between an infection and healthcare facilities reporting a diagnosis, and underestimated as a result of limited access to healthcare or diagnostic capacity.^9^ Once an outbreak is detected, the window for containment may have passed. To avoid waiting for an observable increase in reported cases before undertaking action, the WHO advocates for the integration of threat, hazard and vulnerability data as part of routine, collaborative surveillance.^10^ While hazards are processes or events that impact exposure, infection or disease progression, threats are agents or developments that can affect health security, and vulnerabilities can make individuals or systems susceptible to the effects of hazards.^11^

During the COVID-19 pandemic, enhanced surveillance systems, such as digital contact tracing, genome sequencing, wastewater surveillance or absenteeism monitoring, were set up, but most are being scaled back as resources are scarce and priorities shift.^12^ At the same time, public health authorities reflect on the integration of such enhanced surveillance components with relevance beyond COVID-19, to inform prevention, preparedness and control of other endemic or emerging diseases.^13^

The response to the detection of vaccine-derived poliovirus type 2 (VDPV2) in London sewage in 2022 illustrated the potential of integrated surveillance of threats, hazards and vulnerabilities.^14^ Following repeated detection of the virus in sewage (hazard), subpopulations with insufficient vaccination coverage were identified (vulnerability). Primary care providers then reached out to un(der)vaccinated children for catch-up vaccination. Thus, before a single paralysis case was reported, an anticipated outbreak could be prevented, guided by data on risk (hazard and vulnerability) rather than on disease.

We reviewed successful applications of the monitoring of risks to inform infectious disease prevention and control, and their potential for integration with facility-based surveillance. We then propose low-cost data sources to monitor risks.

## Risk data informing infectious disease outbreak prevention/control

In public health, risk can be (1) exposure, or a proxy for it, such as the aforementioned persisting presence of VDPV2 in sewage indicating viral shedding in the community, potentially exposing susceptible individuals, (2) determinants of exposure, such as recent travel to countries where VDPV2 is circulating, (3) determinants of disease progression, such as insufficient pathogen-specific antibody level as a result of poor vaccination coverage, (4) presymptomatic infection or carriage, such as faecal shedding of VDPV2 and (mis)match with used poliovaccines or (5) characteristics of the pathogen, such as virulence or resistance genes or immune escape of clinical isolates (figure 1). Risk surveillance focuses on the probability of exposure, of infection, and of the transition between stages of disease progression, rather than having a focus on counting cases of each stage.

### One Health risks

Other recent examples of sewage surveillance applications involve metagenomic sequencing of infectious agents and their resistance genes in different One Health compartments. Sequencing samples drawn from sewage or human wastewater plants aims to monitor the presence and/or abundance, before any clinical cases are diagnosed and reported.^15 16^ In the absence of sewage systems, pooled environmental, manure or slaughterhouse waste samples are proposed and piloted as alternatives to track gene presence/abundance across One Health sectors.^17^ Effective integration of such environmental or veterinary sampling as part of food safety, research or for animal health, with human health surveillance, can effectively guide integrated prevention/control interventions, not limited to a single One Health compartment.^17^ During the 2021–2024 surge of highly pathogenic avian influenza A(H5N1), surveillance initially focused on wild and livestock birds, its known reservoir. Following reports of influenza A(H5N1) in dairy cows and two humans after interaction with infected dairy cattle in April 2024, enhanced surveillance is now screening sewage and retail milk for H5N1 RNA. This not only allows for tracking new outbreaks in livestock cattle but also monitors the risk of exposure to humans.^18^

Furthermore, determining and monitoring the spatial distribution of (intermediate) animal hosts (eg, canine rabies)^19^ or vectors (eg, using cumulative *Aedes* mosquito infestation levels or the presence of *Aedes* breeding sites)^20^ can successfully inform geographical targeting of control interventions. Entomological surveys or vector observation in citizen science projects could guide vector control or early warning of emergence to reduce the risk of arbovirus infections such as dengue, chikungunya and zika (table 1).^21^

**Table 1:** Disease and risk indicator (signals) for epidemic intelligence, stages of exposure to disease progression impacted, applied on arbovirus outbreaks (eg, West Nile Virus, dengue, chikungunya and yellow fever)

Disease/risk	Indicator signal	Disease 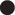 /threat 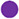 /hazard 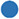 /vulnerability 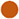	Stage disease progression	Examples of public health measures possibly introduced or scaled up, based on the change in (one or more) disease or risk indicator(s)
**Infection or disease**				
Case number	Single lab confirmed case	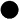	Disease	Outbreak investigation, screening of neighbourhood population
	Increase in incidence	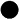	Disease	Targeted awareness campaign, risk group screening, vaccination
	Exceeding healthcare capacity	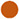	Hospit.	Surge capacity hospital beds
Transmission	Local transmission by endemic vector	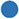	Infect.	Source/contact tracing, quarantine, isolation
	Local transmission from invasive vector	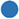	Infect.	Outbreak investigation, quarantine
	Cluster of genomically linked human cases	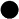	Infect.	Outbreak investigation, link to common source/location
	Time since last reported case	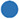	Infect.	Contact tracing, vector control
	Sustained transmission	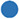	Infect.	Increasing hospital capacity, remove breeding sites, health education
Death number	Single possible/confirmed death	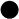	Death	Outbreak investigation; search cases
	Case fatality above a threshold	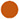	Death	Vaccination; Free healthcare (reduce healthcare seeking delay)
Infection rate	Increased infection rate in subpopulation	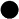 / 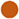	Infect.	(Catch-up) vaccination of at-risk subpopulation
**Onehealth**				
Vector	Presence/abundance of vectorNumber of breeding sites	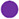 / 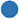 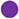	Expos.Expos.	Breeding site removal; public awareness campaign; enhanced disease surveillance
Climatic	Flooding	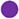	Expos.	
	Average temperature	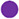	Expos.	
Sewage	Presence/abundance of pathogen or vector genes	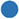	Expos.	Early warning to scale up testing or search for cases or vector
Crowding	Refugee camp	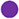 / 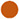	Expos/infect/dis./hospi	Repeated vaccination campaigns; health education
Animal	Livestock/wildlife arbovirus outbreak	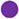 / 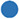	Expos.	Vector control; health education; enhanced disease surveillance
**Behavioural**				
Population mobility	Estimation of imported cases derived from inbound air passenger data, related outbreak risk	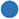	Infect.	Passenger screening or quarantine; travel restrictions; test prior to air travel or at arrival
	Places visited by cases (eg, home, work, school)	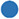	Expos.	Spatially targeted interventions
Absenteeism	Increase in absenteeism from school/work	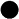	Disease	Syndromic surveillance
Medicine use	Defined daily doses per capita from sales data	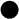	Disease	Syndromic surveillance, if testing capacity limited
**Programmatic**				
Vaccination	Start arbovirus outbreak (for dengue in populations with endemic dengue)	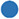	Disease	Vaccination campaign
Health literacy	Awareness role vector breeding sites	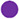	Expos.	Public awareness campaign
Vector control	Number of breeding sites	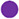 / 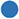	Expos.	Breeding site removal; public awareness campaign
**Population/host factors**				
Malnutrition	Stunting, wasting	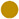	Disease/hospit.	Programmes supporting food security, biodiversity in agriculture
Immunity	Prevalence of (serotype)-specific antibodies	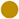	Infect.	Population serological survey; vaccination
Comorbidities	Prevalence immunodeficiency	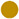	Hospit.	Targeted vaccination
	Sero-prevalence among pregnant women	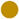	Hospit.	Antenatal care
**Pathogen characteristics**				
Serotyping	Serotyping of dengue virus underlying infection/outbreak	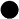	Infect./hospit.	Vaccination campaign; targeted vaccination (in seropositive); voluntary traveller screening programmes (eg, geosentinelles)

### Behaviour

Generally, the occurrence or emergence of an outbreak and its growth rate relate to the rate of contact between hosts or between vectors and hosts, the probability of transmission given a contact, and the mean duration of infectiousness. These are all influenced by host or vector behaviour, including mobility and social network structures. COVID-19 epidemic or pandemic growth—and the effectiveness of physical distancing measures to interrupt contacts and reduce exposure—were estimated from existing mobility and contact data, for example, mobile phones measuring exposure through an exchange of anonymous, randomly generated IDs between phones using Bluetooth,^22^ user data of the National Health Service COVID-19 app^23^ or from the average number of reported contacts during COVID-19 contact tracing.^24^ Activities, locations and subgroups with higher rates of SARS-CoV-2 transmission were identified from mobile phone location data or check-ins on social media.^25^,^27^ Mathematical modelling combined with flight itinerary data has estimated the effect of travel restrictions on imported Ebolavirus disease (EVD) or SARS-CoV-2 cases.^28^,^30^ Despite demonstrations in research context of the usefulness of contact data to predict local outbreak patterns, most countries do not routinely use mobility data as part of outbreak risk assessment or surveillance, nor issued policies on the use of local data sources.

### Health literacy and programmatic data

Dynamic transmission models are increasingly informing pandemic and epidemic responses. Such models, when calibrated with data on the successful uptake of interventions, can—and have—successfully estimated the risk of disease (outbreaks), for example, using data on the (correct) use of insecticide-treated bednets for malaria.^31^ Other such uses of programmatic data could involve condom use for sexually transmitted infections, and handwashing surveys for gastroenteritis, droplet-transmitted respiratory infections, or healthcare-associated infections. Such hazards or vulnerabilities are routinely collected through programmatic data thus readily available for surveillance.

### Population and host factors

Age-specific levels of immunity of the population, estimated directly from serosurveys or indirectly from vaccination coverage, allow estimating the risk of an outbreak of a known pathogen occurring, the expected amplitude of a potential outbreak, its timing, groups most at risk of infection and effectiveness of interventions in mitigating the outbreak. Similarly, the population’s age distribution and the prevalence of comorbidities will determine outbreak size and severity.

Targeting the most at-risk populations can improve interventions’ efficiency and effectiveness. For seasonal or recurring infectious diseases, timing, geographical areas and subpopulations at higher risk can be identified from historic surveillance data, even if prone to reporting bias from differing healthcare utilisation or diagnostic capacity. Several existing interventions target populations at increased risk, based on previously observed spatial or social distribution. In many countries, tuberculosis or multidrug-resistant tuberculosis screening targets specific subpopulations, that is, individuals born in countries with a high background prevalence, household contacts of diagnosed cases and other vulnerable groups.^32^ The global cholera elimination strategy proposes to focus interventions on so-called hotspots, geographically limited areas, based on local case incidence, temporality of transmission, role in spread to other regions or areas, case fatality and vulnerability in terms of access to healthcare, to safe water and sanitation.^33^ For malaria and dengue, hotspots have been defined as transmission foci where individuals and households are at higher risk to be infected, and then transmit the parasite, quantified respectively through population screening and by combined use of vector abundance, human population density and historical cumulative incidence.^20 34^

### Emerging pathogens and changing pathogen characteristics

Throughout EVD, Lassa Fever, COVID-19 and mpox outbreaks, genomic sequencing capacity increased globally, yet most applications are for research purposes, and the use for routine infectious disease surveillance remains limited.^35^ This while integrating genome sequencing within public health surveillance has proven pivotal for the effective support of (1) the detection of emerging infectious agents in humans, animals or environment that could pose a public health threat, that could be translated into target genes for diagnostics, (2) the detection of mutations or genes in existing infectious agents that could alter their virulence, transmissibility, AMR or diagnostic detection, (3) estimating transmissibility,^36^ (4) confirm or discard transmission chains within outbreaks, and (5) confirm or disprove that related cases belong to the same outbreak, improving specificity and timeliness of the detection of outbreaks,^37^ or (6) determine index case or source, informing outbreak investigations and control.

AMR profiles of bacterial pathogens are successfully predicted from metagenomic sequence data of pooled stool and sewage samples, with anticipated potential to guide antimicrobial treatment.^38 39^ Using selective bacterial culture on filtered sewage samples, the Dutch institute for public health and environment monitors population carriage of carbapenemase-producing Enterobacterales, among other applications to determine the role of hospitals as acquisition sources and to monitor the emergence of new resistance genes.^40^

During the 2019 dengue virus outbreak in the Philippines, genetic sequencing was used to identify and track the spread of different dengue virus serotypes, guiding targeted vector control and public health campaigns in the most affected regions. Moreover, vaccination strategies are better informed by serotype-specific vaccine efficacy while varying across serotypes.^41^

## Low-cost data sources to improve risk monitoring beyond facility-based surveillance

### Medical records

To estimate outbreak potential or identify at-risk populations, medical acts (eg, vaccination or absence of it), diagnoses (eg, hypertension) or treatments (eg, antibiotics) can be compiled to infer the risk of infection, disease progression, severity or deaths. Several low-income and middle-income countries (LMICs) have harmonised electronic medical records, using District Health Information Software (DHIS), an open-source health information management system, offering opportunities for disease surveillance without additional workload and resources.^42^ Its continued availability allows monitoring trends in comorbidities, vaccination coverage and medicine use over time and as outbreaks come and go. DHIS data have been used to assess the effect of free healthcare provision on healthcare utilisation during an Ebola outbreak in DR Congo, malaria control interventions in Burundi, for COVID-19 and measles surveillance in the DR Congo, facility-based case reports in Guinea, among several examples.^43^,^46^

### Population cohort-based surveillance

Most resource-constrained countries lack robust civil birth and death registries but have one or more population cohorts or other community-based surveillance networks. Many such systems can provide demographic, socioeconomic, environmental, human host factors or behavioural indicators linked to population denominators.

Health and Demographic Surveillance Systems (HDSSs) consist of a longitudinal follow-up of a population cohort living in a well-defined geographical area, regularly registering largely similar data—mainly for research purposes. By systematically collecting households’ demographic, health status and risk data during household visits or phone calls, HDSS could resolve important limitations of facility-based disease surveillance. HDSS could function as a network of sentinel populations to monitor risks, infer measures of disease occurrence using accurate denominators and healthcare seeking behaviours and evaluate the performance of interventions or populations’ susceptibility (embedding serosurveys).

Participatory surveillance, in which volunteers in a population cohort can participate and report data, could be a stand-alone, less resource-demanding alternative or complement to population-based surveillance. Telephone applications can facilitate self-reporting of behaviour, exposures and symptoms by surveillance participants. Thus, reported syndromes could support outbreak detection and reported behaviour or sentiments could inform practices associated with disease, similar to InfluenzaNet since 2003 in the Netherlands (now called infectieradar), monitoring attitudes towards COVID-19 vaccination, testing and isolation in Italy, France and Belgium and the ZOE COVID app in the UK.^47^,^49^ Nasal self-swabs from randomly selected volunteers sent by mail in the UK indicated SARS-CoV-2 infection prevalence, anticipating increases in hospital admissions and deaths.^50^ Several countries established in recent years initiatives involving the public to count and report mosquitos.^51^

### Repeated cross-sectional studies

A national health survey was conducted in 1935 in the USA, as a way to obtain risk and morbidity data with reliable population denominators.^52^ Since the 1980s and 90s, repeated health surveys have been established in numerous LMICs under different formats. The Demographic and Health Surveys (DHSs) and the Multiple Indicator Cluster Surveys demonstrated how household surveys can provide trends in population’s or patients’ demographic and health indicators in order to evaluate public health interventions. Use of data generated from these surveys by public health institutions, officials and researchers is, however, limited or delayed,^53^ even though they could provide information on relevant risk indicators such as hand-washing practices, vaccination coverage, bednet use, health status or healthcare utilisation. Demographics and healthcare utilisation reported in DHS could be used, respectively, to provide the population structure for mathematical infectious disease models and to infer the actual number of disease cases from the limited number of cases that sought healthcare.

Repeated outpatient or hospital-based point-prevalence surveys can provide important and low-cost surveillance on clinical presentations, duration of admission, antibiotic use, AMR, quality of care and infection prevention and control. Point-prevalence surveys are widely used globally to identify outbreaks of healthcare-associated infections and estimate their prevalence.^54^

### Medicine distribution

In countries where the production and import of medicines is traced, sales data or records of the amount of active ingredient consumed can be used to estimate nationwide medicine use, particularly antibiotic use.^55^ Health insurance data have been used to monitor disease incidence and to estimate testing rates for sexually transmittable diseases, thus continuously evaluating the control strategy’s effectiveness.^56^

## Outlook to future outbreak control

Technological and diagnostic advances can facilitate embedding risks in infectious disease surveillance. Digital disease and risk data are more rapidly available, as are non-traditional digital data sources of which some listed above (eg, mobility data), but also OpenStreetMap, traditional and social media have increasingly been leveraged Initiatives such as Global.health automatically collate and curate standardised, deidentified case records at the start of emerging disease outbreaks.^57^ Standardising data collection over time and across countries and regions allows for improved multicountry analyses, in addition to (semi)automising predictable tasks related to disease surveillance and control, that is, data cleaning processes, linkage of disease and risk data, efficient analysis and signal detection. The use of large language models, either directly (eg, prompting technology to produce computer code) or indirectly by setting up agents that communicate on dedicated tasks, could support data and intelligence pipeline implementation even if resources are constrained.^58^ Over time, experience gained through the detection of signals subsequently investigated will allow establishment and fine-tuning of risk indicator thresholds. As the association between risk and disease or outbreaks is not by definition linear over time, place and populations, risk data require careful and continuous validation. Timely data interoperability, linkage and data sharing at the start of an outbreak require clear governance and responsibilities for who generates and who uses data and should be decided prior to an outbreak occurring.^59^

Increasing availability of point-of-care molecular or lateral flow tests, currently already widely used in many countries for tuberculosis, malaria or COVID-19, and the integration of test results provide dual advantages of improved clinical diagnosis and early detection of cases and outbreaks.

Better collaboration on surveillance across disease and threat surveillance systems, across sectors, emergency cycles and geographical levels, as propagated by the WHO,^10^ could (1) speed up signal detection, (2) better identify populations at increased risk of infection, disease, hospitalisation or death, through linkage of risk, disease, diagnostic and population data, (3) improve case ascertainment, by regularly updating case definitions to the latest epidemiological situation, including geographical foci with pathogen circulation, occupational risks, setting-specific transmission or a pathogen’s AMR profile. Targeted interventions, differentiating between risk profiles, will be more effective, (cost)efficient and acceptable than generic interventions based on facility-based clinical data only.

## Conclusion

Infectious disease surveillance is increasingly moving beyond facility-based case reporting, offering opportunities for more timely, more effective, better targeted and adapted infectious disease prevention and control measures and epidemic preparedness. Countries and regions need to identify and prioritise which threats, hazards and vulnerabilities can best inform their prevention and control measures, how data can be made available, regularly analysed, what intelligence should be generated from the data, when and by whom, and how intelligence should be disseminated for disease prevention and control.

## Data Availability

Data sharing not applicable as no datasets generated and/or analysed for this study.

## References

[1] Smith KF, Goldberg M, Rosenthal S (2014). Global rise in human infectious disease outbreaks. J R Soc Interface.

[2] Jones KE, Patel NG, Levy MA (2008). Global trends in emerging infectious diseases. Nature New Biol.

[3] Bernstein AS, Ando AW, Loch-Temzelides T (2022). The costs and benefits of primary prevention of zoonotic pandemics. Sci Adv.

[4] Neiderud CJ (2015). How urbanization affects the epidemiology of emerging infectious diseases. Infect Ecol Epidemiol.

[5] Bedford J, Farrar J, Ihekweazu C (2019). A new twenty-first century science for effective epidemic response. Nature New Biol.

[6] Blauer B, Brownstein JS, Gardner L (2023). Innovative platforms for data aggregation, linkage and analysis in the context of pandemic and epidemic intelligence. Euro Surveill.

[7] Kraemer MUG, Sinka ME, Duda KA (2015). The global distribution of the arbovirus vectors Aedes aegypti and Ae. albopictus. *Elife*.

[8] Carlson CJ, Albery GF, Merow C (2022). Climate change increases cross-species viral transmission risk. Nature New Biol.

[9] World Health Organization (2018). Antimicrobial resistance and primary health care.

[10] Archer BN, Abdelmalik P, Cognat S (2023). Defining collaborative surveillance to improve decision making for public health emergencies and beyond. Lancet.

[11] World Health Organization (WHO) (2023). Defining collaborative surveillance. A core concept for strengthening the global architecture for health emergency preparedness, response, and resilience (HEPR).

[12] Eales O, Plank MJ, Cowling BJ (2024). Key Challenges for Respiratory Virus Surveillance while Transitioning out of Acute Phase of COVID-19 Pandemic. Emerg Infect Dis.

[13] World Health Organization (WHO) (2023). 'Crafting the mosaic': a framework for resilient surveillance for respiratory viruses of epidemic and pandemic potential.

[14] UK Health Security Agency (2022). Poliovirus detected in sewage from north and east london.

[15] Aarestrup FM, Woolhouse MEJ (2020). Using sewage for surveillance of antimicrobial resistance. Science.

[16] Hendriksen RS, Munk P, Njage P (2019). Global monitoring of antimicrobial resistance based on metagenomics analyses of urban sewage. Nat Commun.

[17] Aarestrup FM, Bonten M, Koopmans M (2021). Pandemics– One Health preparedness for the next. *The Lancet Regional Health - Europe*.

[18] Wallace HL, Wight J, Baz M Longitudinal influenza a virus screening of retail milk from canadian provinces (rolling updates). *Infectious Diseases (except HIV/AIDS*).

[19] Velasco-Villa A, Escobar LE, Sanchez A (2017). Successful strategies implemented towards the elimination of canine rabies in the Western Hemisphere. Antiviral Res.

[20] Baldoquín Rodríguez W, Mirabal M, Van der Stuyft P (2023). The Potential of Surveillance Data for Dengue Risk Mapping: An Evaluation of Different Approaches in Cuba. *Trop Med Infect Dis*.

[21] Deblauwe I, Brosens D, De Wolf K (2022). MEMO: Monitoring of exotic mosquitoes in Belgium. GigaByte.

[22] Abueg M, Hinch R, Wu N (2021). Modeling the effect of exposure notification and non-pharmaceutical interventions on COVID-19 transmission in Washington state. NPJ Digit Med.

[23] Wymant C, Ferretti L, Tsallis D (2021). The epidemiological impact of the NHS COVID-19 app. Nature New Biol.

[24] Ingelbeen B, Peckeu L, Laga M (2021). Reducing contacts to stop SARS-CoV-2 transmission during the second pandemic wave in Brussels, Belgium, August to November 2020. Euro Surveill.

[25] Chang S, Pierson E, Koh PW (2021). Mobility network models of COVID-19 explain inequities and inform reopening. Nature New Biol.

[26] Levin R, Chao DL, Wenger EA (2021). Insights into population behavior during the COVID-19 pandemic from cell phone mobility data and manifold learning. *Nat Comput Sci*.

[27] Worobey M, Levy JI, Malpica Serrano L (2022). The Huanan Seafood Wholesale Market in Wuhan was the early epicenter of the COVID-19 pandemic. Science.

[28] Poletto C, Gomes MF, Pastore y Piontti A (2014). Assessing the impact of travel restrictions on international spread of the 2014 West African Ebola epidemic. Euro Surveill.

[29] Bogoch II, Watts A, Thomas-Bachli A (2020). Pneumonia of unknown aetiology in Wuhan, China: potential for international spread via commercial air travel. J Travel Med.

[30] Wu JT, Leung K, Leung GM (2020). Nowcasting and forecasting the potential domestic and international spread of the 2019-nCoV outbreak originating in Wuhan, China: a modelling study. Lancet.

[31] Sherrard-Smith E, Winskill P, Hamlet A (2022). Optimising the deployment of vector control tools against malaria: a data-informed modelling study. Lancet Planet Health.

[32] Trauer JM, Dodd PJ, Gomes MGM (2019). The Importance of Heterogeneity to the Epidemiology of Tuberculosis. Clin Infect Dis.

[33] Global Task Force on Cholera Control (2017). Ending cholera. A global roadmap to 2030.

[34] Bousema T, Drakeley C, Gesase S (2010). Identification of Hot Spots of Malaria Transmission for Targeted Malaria Control. J INFECT DIS.

[35] Inzaule SC, Tessema SK, Kebede Y (2021). Genomic-informed pathogen surveillance in Africa: opportunities and challenges. Lancet Infect Dis.

[36] Volz E, Mishra S, Chand M (2021). Assessing transmissibility of SARS-CoV-2 lineage B.1.1.7 in England. Nature New Biol.

[37] Tang P, Croxen MA, Hasan MR (2017). Infection control in the new age of genomic epidemiology. Am J Infect Control.

[38] Auguet OT, Niehus R, Gweon HS (2021). Population-level faecal metagenomic profiling as a tool to predict antimicrobial resistance in *Enterobacterales* isolates causing invasive infections: An exploratory study across Cambodia, Kenya, and the UK. EClinicalMedicine.

[39] Munk P, Brinch C, Møller FD (2022). Genomic analysis of sewage from 101 countries reveals global landscape of antimicrobial resistance. Nat Commun.

[40] Blaak H, Kemper M, De RA (2023). Wastewater based surveillance of AMR in the Netherlands.

[41] Paz-Bailey G, Adams LE, Deen J (2024). Dengue. Lancet.

[42] Hazel E, Wilson E, Anifalaje A (2018). Building integrated data systems for health and nutrition program evaluations: lessons learned from a multi-country implementation of a DHIS 2-based system. J Glob Health.

[43] Reynolds E, Martel LD, Bah MO (2022). Implementation of DHIS2 for Disease Surveillance in Guinea: 2015–2020. Front Public Health.

[44] Hategeka C, Carter SE, Chenge FM (2021). Impact of the COVID-19 pandemic and response on the utilisation of health services in public facilities during the first wave in Kinshasa, the Democratic Republic of the Congo. *BMJ Glob Health*.

[45] Hung YW, Law MR, Cheng L (2020). Impact of a free care policy on the utilisation of health services during an Ebola outbreak in the Democratic Republic of Congo: an interrupted time-series analysis. *BMJ Glob Health*.

[46] Cellule d’Analyses Intégrées (2022). Analyse opérationnelle sur les dynamiques autour de l’épidémie de rougeole à kinshasa: synthèse des résultats clefs et des recommandations - democratic republic of the congo | reliefweb.

[47] Marquet RL, Bartelds AIM, Noort SP (2006). Internet-based monitoring of influenza-like illness (ILI) in the general population of the Netherlands during the 2003-2004 influenza season. BMC Public Health.

[48] de Meijere G, Valdano E, Castellano C (2023). Attitudes towards booster, testing and isolation, and their impact on COVID-19 response in winter 2022/2023 in France, Belgium, and Italy: a cross-sectional survey and modelling study. *The Lancet Regional Health - Europe*.

[49] Menni C, Valdes AM, Polidori L (2022). Symptom prevalence, duration, and risk of hospital admission in individuals infected with SARS-CoV-2 during periods of omicron and delta variant dominance: a prospective observational study from the ZOE COVID Study. *The Lancet*.

[50] Pouwels KB, House T, Pritchard E (2021). Community prevalence of SARS-CoV-2 in England from April to November, 2020: results from the ONS Coronavirus Infection Survey. Lancet Public Health.

[51] Sciensano (2022). MuggenSurveillance. een burgerplatform voor toezicht op en rapportering over de tijgermug in belgië. https://muggensurveillance.be/.

[52] Thacker SB, Qualters JR, Lee LM (2012). Public health surveillance in the United States: evolution and challenges. MMWR Suppl.

[53] Huang Y, Danovaro-Holliday MC (2021). Characterization of immunization secondary analyses using demographic and health surveys (DHS) and multiple indicator cluster surveys (MICS), 2006-2018. BMC Public Health.

[54] Suetens C, Latour K, Kärki T (2018). Prevalence of healthcare-associated infections, estimated incidence and composite antimicrobial resistance index in acute care hospitals and long-term care facilities: results from two European point prevalence surveys, 2016 to 2017. Euro Surveill.

[55] Klein EY, Milkowska-Shibata M, Tseng KK (2021). Assessment of WHO antibiotic consumption and access targets in 76 countries, 2000-15: an analysis of pharmaceutical sales data. Lancet Infect Dis.

[56] Viriot D, Lucas E, Barbeyrac B (2006). Use of healthcare reimbursement data to monitor bacterial sexually transmitted infection testing in France. Euro Surveill.

[57] Kraemer MUG, Tegally H, Pigott DM (2022). Tracking the 2022 monkeypox outbreak with epidemiological data in real-time. Lancet Infect Dis.

[58] van Hoek AJ, Funk S, Flasche S (2024). Importance of investing time and money in integrating large language model-based agents into outbreak analytics pipelines. Lancet Microbe.

[59] Bogaert P, Verschuuren M, Van Oyen H (2021). Identifying common enablers and barriers in European health information systems. Health Policy.

